# Aquatic circoviruses: emerging pathogens in global aquaculture — from discovery to disease management

**DOI:** 10.1128/jvi.01737-24

**Published:** 2024-12-13

**Authors:** Yuanyuan Wang, Changjun Guo, Jianguo He

**Affiliations:** 1 School of Marine Sciences, State Key Laboratory for Biocontrol, Southern Marine Science and Engineering Guangdong Laboratory (Zhuhai), Guangdong Province Key Laboratory for Aquatic Economic Animals & Guangdong Provincial Observation and Research Station for Marine Ranching of the Lingdingyang Bay, Sun Yat-sen University 373651 , Guangzhou, China; Universiteit Gent, Merelbeke, Belgium

**Keywords:** aquaculture, circovirus, virology, pathology, epidemiology, metagenomics

## Abstract

The expansion of global aquaculture has brought challenges from emerging pathogens, resulting in disease-related production losses across various regions. Among these pathogens, aquatic circoviruses—small, single-stranded DNA viruses initially detected in barbel (*Barbus barbus*)—have now been identified in multiple aquaculture species. These viruses have been associated with various clinical manifestations in economically important fish, crustacean, and mollusk species, including acute hemorrhage syndrome, which has shown mortality rates up to 95% in controlled laboratory infections of turbot. This review consolidates current knowledge on aquatic circoviruses, focusing on their genetic diversity, epidemiology, pathogenesis, and management strategies. The analysis encompasses observed host range patterns, documented instances of cross-species transmission, and evolutionary characteristics, such as host-specific clustering and recombination events. Research gaps are highlighted, particularly in understanding viral pathogenic mechanisms, host–pathogen interactions, and their ecological roles within aquatic ecosystems. We evaluate recent advances in diagnostic methods, including targeted vaccine design and RNA interference technology. The review outlines future research priorities, including elucidating cross-species transmission potential, developing effective treatments, and assessing the full economic impact of these viruses on aquaculture. By providing a comprehensive overview, this review aims to guide future research efforts and inform strategies to mitigate the impact of circoviruses on aquaculture sustainability.

## INTRODUCTION

Aquaculture represents the most rapidly expanding sector in food production, playing an instrumental role in addressing the escalating global demand for protein-based nutrition. According to the Food and Agriculture Organization, aquaculture now supplies over 50% of the global fish consumption, with a market value exceeding $472 billion in 2022 (1). However, the industry faces mounting challenges from emerging diseases, primarily attributed to the international movement of live organisms, intensification of farming practices, and increasing anthropogenic stressors on aquatic ecosystems (2). This rapid expansion and intensification have created optimal conditions for the emergence and spread of viral pathogens (3).

Within this context, circoviruses have emerged as significant pathogens, belonging to a diverse family of small, single-stranded DNA viruses that can infect a wide range of hosts, including mammals, birds, fish, and invertebrates (4). Their detection in aquaculture systems has garnered substantial attention due to the potential economic impact on commercially valuable species. The initial discovery of fish circoviruses in barbel (*Barbus barbus*) from Hungarian waters in 2011, followed by their detection in European catfish (*Silurus glanis*) in 2012 (5, 6), marked a paradigmatic shift in our understanding of circovirus ecology, as these pathogens were historically associated predominantly with terrestrial hosts. Subsequent investigations have revealed circovirus presence in diverse fish species, notably including turbot (*Scophthalmus maximus*) (7). The identification of turbot circovirus (TurCV) has raised significant concerns due to its associated mortality rates and potential economic impact (7). Research indicates that these viruses exhibit broader distribution patterns in aquatic ecosystems than previously recognized, with their molecular characteristics—notably their small size and circular genome structure—potentially facilitating environmental persistence and transmission (8, 9).

Circoviruses pose unique challenges in aquaculture due to their considerable genetic diversity, broad host range, and environmental stability (8). Recent advancements in metagenomic sequencing have revealed numerous novel circovirus variants in aquatic species, underscoring their prevalence and genetic heterogeneity in both marine and freshwater environments (9). Although the pathogenesis of aquatic circoviruses remains incompletely characterized, studies in terrestrial animals, particularly pigs, have linked circoviruses to mortality and significant economic losses (10). Hence, there is a need for further research into the potential economic and ecological impacts of aquatic circoviruses on aquaculture.

This comprehensive review synthesizes current knowledge regarding aquaculture-associated circoviruses, encompassing their genetic diversity, epidemiological patterns, pathogenic mechanisms, diagnostic approaches, and control strategies. Through critical analysis of existing research, we aim to identify knowledge gaps and prioritize future research directions to enhance our understanding and management of these emerging pathogens in aquaculture systems.

## GENETICS AND MOLECULAR BIOLOGY

### Genotyping and genome structure

The significance of circoviruses in aquaculture has become increasingly apparent through their identification in diverse economically important aquatic species. Initially documented in *B. barbus* fry exhibiting high mortality rates (5), circovirus infections have subsequently been detected across a broad spectrum of cultivated aquatic organisms (7, 9, 11). These viruses exhibit exceptional host diversity, infecting organisms across multiple phyla in both marine and freshwater environments globally. Their host ranges encompass *Actinopterygii* (bony fish), *Chondrichthyes* (cartilaginous fish), *Crustacea*, and *Mollusca*. Expanding upon the foundational work of Varsani et al. (12), we provide an updated compilation of aquatic circoviruses and circovirus-related viruses in (Table S1), incorporating recent discoveries from a wide range of aquatic taxa.

Advanced metagenomic analyses have revealed unprecedented circovirus diversity in crustaceans, exemplified by the discovery of a novel circovirus-like genome in *Farfantepenaeus duorarum* hepatopancreas from the Gulf of Mexico (13). The first documented mollusk-associated circovirus genome was identified in *Amphibola crenata* (14), with subsequent detections in commercially significant bivalve species (9), particularly through comprehensive virome analyses of oyster populations (15).

Genetic characterization has revealed substantial diversity within aquatic circoviruses, with distinct genotypes identified across species. For instance, two distinct genotypes of *European catfish* circovirus have been reported based on phylogenetic analysis of complete genome sequences (6).

**Fig 1:**
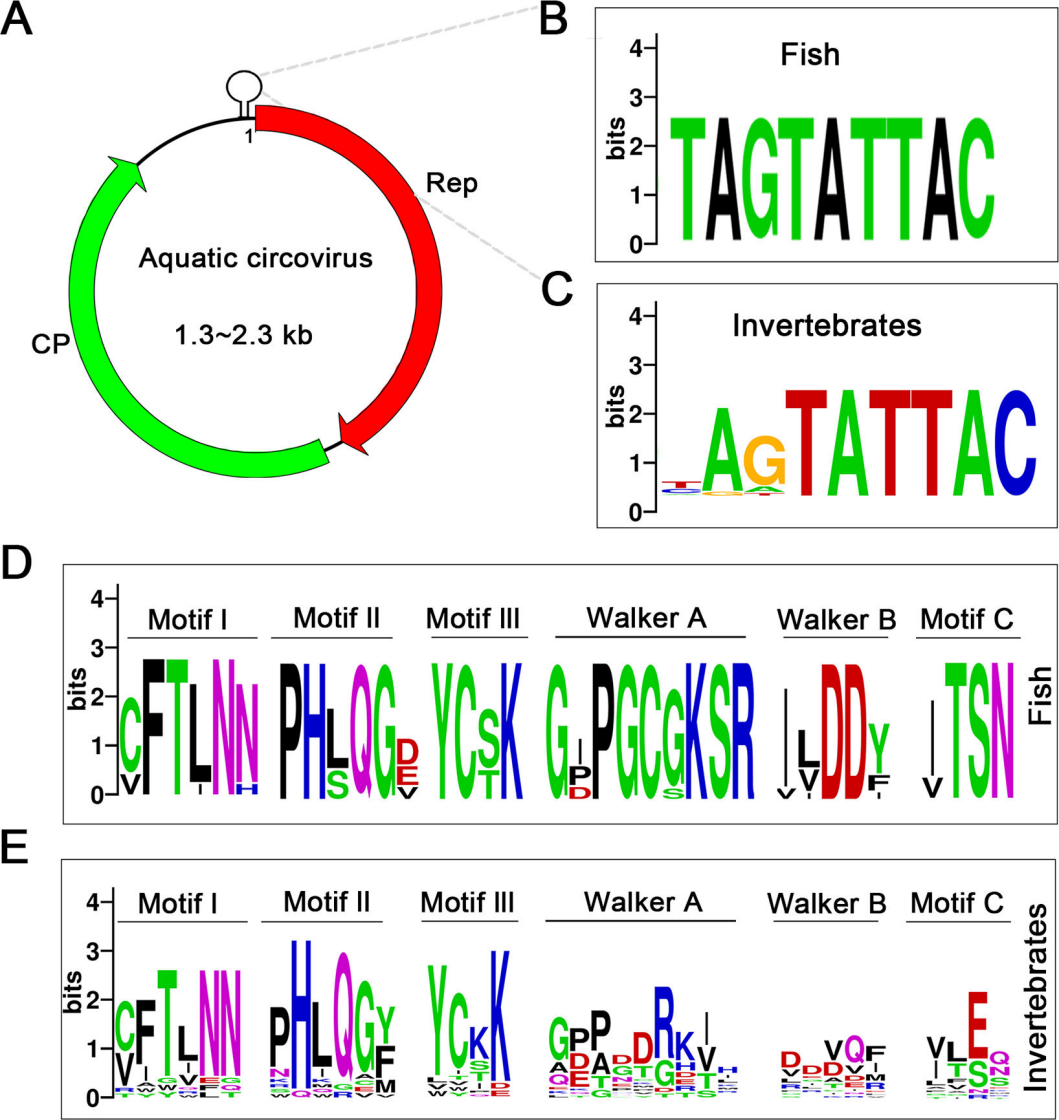
Genomic structure and sequence motifs in fish and invertebrates-associated circovirus. (**A**) The circular ssDNA genome contains two major open reading frames (ORFs) encoding the replication-associated protein (Rep) and capsid protein (CP). A conserved stem-loop structure containing the origin of replication is located in the intergenic region. (**B**) Nonanucleotide motif sequences of fish-associated circovirus. (**C**) Nonanucleotide motif sequences of invertebrate circovirus. (**D**) Conserved amino acid residues in replication-associated proteins (Rep) of fish-associated circovirus. (**E**) Conserved amino acid residues in replication-associated proteins (Rep) of invertebrate circovirus. Panels C and D show sequence probability logos (generated using Weblogo [16]) of conserved amino acid residues characteristic of rolling-circle replication (RCR) endonucleases, including RCR motifs I, II, III, and superfamily 3 helicase motifs, including Walker A and B, motif C. The overall height of the stack indicates the sequence conservation at that position, while the height of symbols within the stack indicates the relative frequency of each amino or nucleic acid at that position. Bits (y-axis) indicate information content at each position. Panels A and B show nucleotide sequences, and C and D show amino acid sequences. Colors represent different nucleotides or amino acids.

The genomic architecture of aquatic circoviruses consists of a covalently closed, circular, single-stranded DNA molecule ranging from 1.3 to 2.3 kb (Table 1). This ambisense genome encodes minimally two essential open reading frames (ORFs): ORF1, encoding replication-associated proteins (Rep), and ORF2, encoding capsid proteins (CP) of 144–341 amino acids (Fig. 1A; Table 1). Additional ORFs have been identified in some circoviruses, but their functions remain largely unknown.

**TABLE 1:** Characteristics of aquaculture circovirus genome

Host class	Circovirus name	Isolation source	Geographical origin	Accession no.	Genome (nt)	Rep (aa)	CP (aa)	Stem loop	Ref.
Fish	Circovirus barbel	Barbel tissue	Hungary	GU799606	1,957	319	166	TAGTATTAC	(5)
	Circovirus barbel	Barbel tissue	Hungary	JF279961	1,957	319	214	TAGTATTAC	(5)
	Circovirus catfish	*Silurus glanis*	Hungary	JO011377	1,966	314	227	TAGTATTAC	(6)
	Circovirus catfish	*Silurus glanis*	Hungary	JQ011378	1,966	314	227	TAGTATTAC	(6)
	Circovirus eel^*a*^	*Anguilla anguilla*	Hungary	KU951578	1,378	286	114	TAGTATTAC	(17)
	Circovirus eel^*a*^	*Anguilla anguilla*	Hungary	KU951579	1,378	286	114	TAGTATTAC	(17)
	Circovirus eel^*a*^	*Anguilla anguilla*	Hungary	KU951580	1,975	286	123	TAGTATTAC	(17)
	Circovirus eel^*a*^	*Anguilla anguilla*	Hungary	NC_023421	1,378	286	114	TAGTATTAC	(11)
	Circovirus Lhasa^*a*^	Fish in Lhasa River	China	OP933698	1,679	292	194	-	-
	Circovirus Turbot^*a*^	*Scophthalmus maximus*	China	PP417825	1,773	310	231	TAGTATTAC*^c^	(7)
Shrimp	Circovirus duorarum^*a*^	*Farfantepenaeus duorarum* (hepatopancreas)	USA	KC441518	1,955	287	181	AGGTATTAC	(13)
	Circovirus monodon^*a*^	*Penaeus monodon*	Vietnam	KF481961	1,777	266	255	TAATATTAC	(18)
	*S.brevirostris* Brown Rock Shrimp aCV^*b*^	*Sicyonia brevirostris* (gonads)	USA	KR528567	1,600	227	193	TAATATTAC^∗^	(9)
	*Palaemonete* sp. Common Grass Shrimp aCV^*b*^	*Palaemonete sp*. (hepatopancreas)	USA	KR528568	2,257	321	297	TAGTATTAC	(9)
	*P. intermedius* Brackish Grass Shrimp aCV^*b*^	*Palaemonetes intermedius*	USA	KR528551	2,293	301	339	CAGTATTAC	(9)
	*F.duorarum* Pink Shrimp aCV^*b*^	*Farfantepenaeus duorarum*	USA	KR528552	1,799	175	288	CAGTATTAC	(9)
	*F.duorarum* Pink Shrimp aCV^*b*^	*Farfantepenaeus duorarum*	USA	KR528553	1,966	305	311	CAGTATTAC	(9)
	*P.kadiakensis* Mississippi Grass Shrimp aCV^*b*^	*Palaemonetes kadiakensis*	USA	KR528560	1,895	305	330	None	(9)
	Gammarus sp. Amphipod aCV^*b*^	*Gammarus* sp.	USA	KR528561	1,999	294	262	TAGTATTAC	(9)
Crab	*Petrochirus diogenes* giant hermit crab aCV^*b*^	*Petrochirus diogenes* (abdomen)	USA	KR528543	1,815	349	226	TAGTATTAC	(9)
	*C.ornatus* Ornate Blue Crab aCV^*b*^	*Callinectes ornatus* (gonads)	USA	KR528549	1,241	264	198	CAGTATTAC	(9)
	*C.sapidus* Atlantic Blue Crab aCV^*b*^	*Callinectes sapidus* (gonads)	USA	KR528550	1,876	270	143	CAGTATTAC	(9)
	Hermit Crab aCV^*b*^	Hermit Crab (abdomen)	USA	KR528555	2,291	304	285	TAGTATTAC	(9)
	Hermit Crab aCV^*b*^	Hermit Crab (abdomen)	USA	KR528556	2,291	304	321	TAGTATTAC	(9)
	Hermit Crab aCV^*b*^	Hermit Crab (abdomen)	USA	KR528557	1,063	288	/	CAGTATTAC	(9)
	Fiddler Crab aCV^*b*^	Fiddler Crab (gonads and claw muscle)	USA	KR528558	1,635	273	227	GATTATTAC	(9)
	Fiddler Crab aCV^*b*^	Fiddler Crab (gonads and claw muscle)	USA	KR528559	1,511	176	143	AAGTATTAC	(9)
Snail	Marine Snail aCV^*b*^	Marine Snail	USA	KR528554	2,305	315	341	TAGTATTAC	(9)
	*Littorina* sp. Snail aCV^*b*^	*Littorina* sp.	USA	KR528548	2,237	366	265	CAGTATTAC	(9)
	Gastropod associated circular ssDNA virus	*Amphibola crenata*	New Zealand	KC172652	2,357	291	318	CAGTATTAC	(14)
Clam	*Mytilus* sp. Clam aCV^*b*^	*Mytilus* sp.	USA	KR528562	1,894	301	284	TAGTATTAC	(9)

^*a*^ Circovirus name is proposed in this article based on the ICTV naming method for virus-related species.

^*b*^ Previous species names contain abbreviation aCV for associated circular virus or aCG for associated circular genome.

^*c*^ Nonanucleotide motif sequences that were not identified within a stem-loop structure are denoted with an asterisk (∗).

A defining genomic feature is the conserved stem-loop structure containing a nonanucleotide motif (NANTATTAC) positioned between the 5′ termini of the major ORFs, serving as the origin of replication (19–21). Fish-associated circoviruses exhibit strong conservation of the “TAGTATTAC” motif, suggesting selective pressure for maintaining replication efficiency. Invertebrate-associated circoviruses display slight variations, such as “AGTATTAC,” potentially reflecting host-specific adaptations (Fig. 1B and C). Comparative analysis of circoviruses that infect terrestrial hosts, including porcine circovirus (PCV) and beak and feather disease virus, reveals remarkable conservation of these motifs across evolutionary lineages, indicating their fundamental role in viral replication mechanisms regardless of host context or environmental conditions (20).

### Structure and function of the encoded proteins

Molecular analysis of replication-associated protein (Rep) proteins from aquatic circovirus reveals distinct conservation patterns within their specific functional domains. Structurally, these proteins feature an N-terminal HUH superfamily endonuclease domain and a C-terminal superfamily three helicase domain, as predicted by comparative analyses with well-studied circoviruses (12). Fish-associated circoviruses retain conserved Rep motifs that are characteristic of the *Circoviridae*, including rolling-circle replication (RCR) motifs like “(C/v)FT(L/I)NN” (motif I) and “G(*P*/x)(*P*/x)GxGK(S/t)” (Walker A motif) (Fig. 1D and E). In these motifs, “x” can represent any amino acid residue, while residues in lowercase are less frequently observed (21). The conservation of these sequences across diverse host species underscores their significant role in viral replication mechanisms and evolution, making them potential targets for therapeutic interventions.

While core motifs demonstrate conservation across both fish-associated and invertebrate circoviruses, notable variations exist, particularly in the Walker A motif of invertebrate-associated viruses, suggesting host-specific adaptations. This sequence plasticity likely reflects distinct evolutionary pressures, exemplified by increased variability in SF3 motifs among invertebrate circoviruses, potentially facilitating broader host range adaptation or environmental resilience.

**Fig 2:**
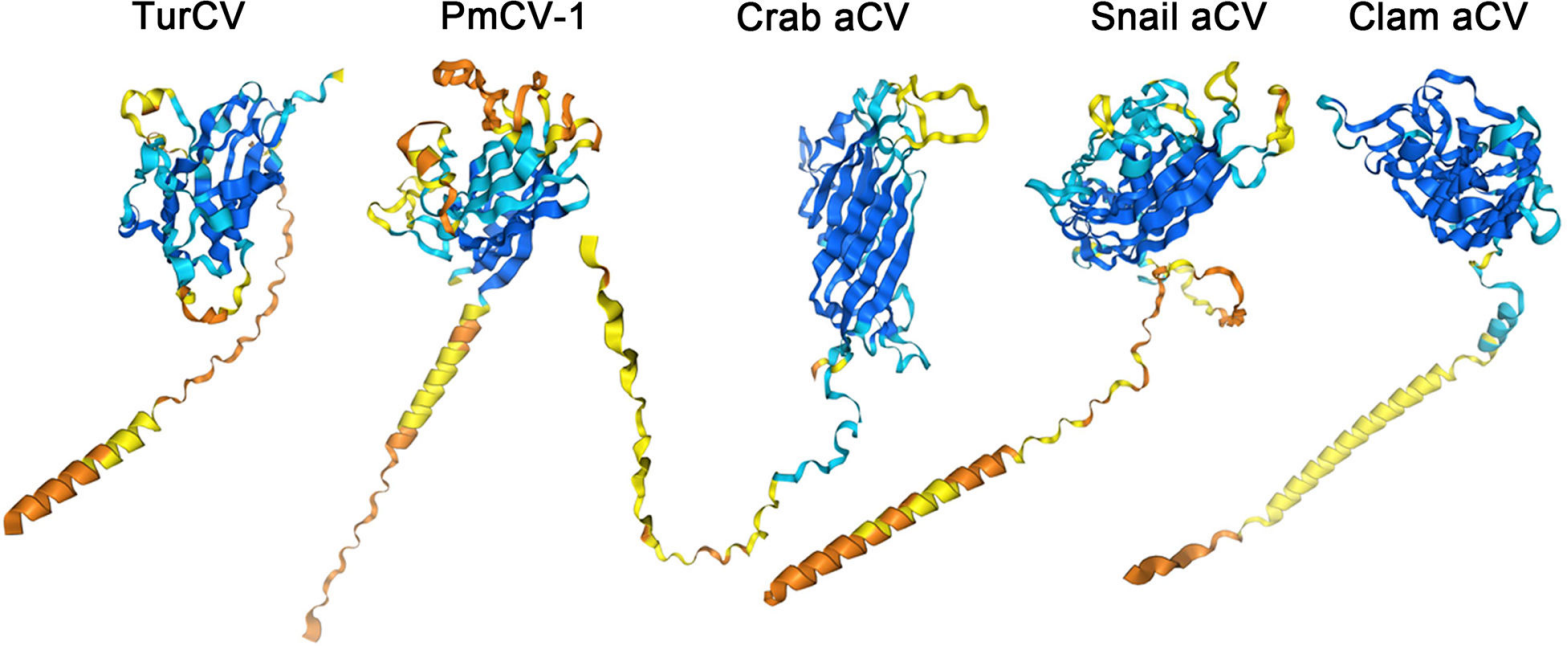
The predicted three-dimensional structures of CP proteins from five representative aquatic circoviruses. TurCV (Turbot circovirus; PP417825), PmCV-1 (Penaeus monodon circovirus-1; KF481961), Crab aCV (Hermit Crab circular virus; KR528555), Snail aCV (Marine Snail circular virus; KR528554), Clam aCV (*Mytilus* sp. circular virus; KR528562). These structures were predicted using AlphaFold3 (24).

The CP protein, however, exhibits greater variability and participates in viral encapsidation and host cell entry. The amino acid sequences of the CP proteins in aquatic circoviruses show significant variation (Fig. S1). However, the reasons for such extensive differences in CP sequences remain unclear. Computational analyses suggest similarities in the CP protein structure among the five representative aquatic circoviruses, consisting of a globular domain (likely the primary capsid structure) and an extended tail region (Fig. 2). The length and conformation of the tail region vary among viruses, suggesting possible functional differences or adaptations. Despite these structural similarities, there are notable differences in the detailed structure, particularly in the precise arrangement of the ring region and secondary structural elements. The capsid assembly follows T = 1 icosahedral symmetry, with 60 CP copies forming isometric capsids (22, 23). Each CP contains a positively charged N-terminal arm followed by a jelly-roll fold domain, consistent with other circoviruses.

### Functional implications of genetic diversity

The observed genetic diversity in aquatic circoviruses has significant functional implications, particularly regarding host range and viral adaptation. The pronounced variability in CP sequences, exceeding that observed in porcine circovirus 2 (PCV2), suggests diverse host interaction mechanisms and presents challenges for broad-spectrum vaccine development.

The relative conservation of Rep proteins across aquatic and terrestrial circoviruses reflects their important role in viral replication. However, variations in SF3 helicase motifs, particularly pronounced in invertebrate circoviruses, may influence replication efficiency and host adaptation. Notably, aquatic circoviruses possess larger genomes (1.9–2.5 kb) compared with PCV2 (1.7–1.8 kb) and demonstrate greater sequence diversity, potentially reflecting adaptations to aquatic environments. These genomic characteristics may confer enhanced environmental stability or expanded host range capabilities, though the precise functional implications await experimental validation.

## EPIDEMIOLOGY

### Susceptible species and transmission routes

The emergence of fish circoviruses was first documented in Europe, with initial detections in Hungarian barbel and European catfish populations (5, 6). Later studies identified circovirus infections in European eel (*Anguilla anguilla*) in the Netherlands (11, 17), followed by identifications in *Scophthalmus maximus* populations in China (7). This broad detection pattern across diverse taxonomic groups suggests considerable potential for cross-species transmission, though the molecular determinants facilitating host adaptation remain to be elucidated.

Aquatic circoviruses exhibit complex patterns of host range specificity and tissue tropism, with viral detection occurring across multiple host species and tissue types (7, 9). A notable epidemiological feature is the age-dependent susceptibility pattern, characterized by elevated detection rates in juvenile populations (25). This age-related vulnerability may stem from the combination of immature immune systems and enhanced cellular proliferation rates in younger animals, creating optimal conditions for viral replication.

Environmental and host physiological factors appear to modulate infection dynamics, particularly in aquaculture environments. High stocking densities, poor water quality, and concurrent infections have been associated with increased viral detection rates. However, the exact causal relationships between these environmental stressors and infection outcomes need rigorous experimental validation. Current research suggests several potential transmission routes for aquatic circoviruses, including vertical transmission from broodstock to offspring, horizontal transmission through water-borne exposure, mechanical transmission *via* contaminated equipment, and potential involvement of biological vectors or intermediate hosts. However, the relative importance of each transmission route and the specific mechanisms underlying them necessitate detailed experimental elucidation.

### Ancestral tracing of aquatic circoviruses

**Fig 3:**
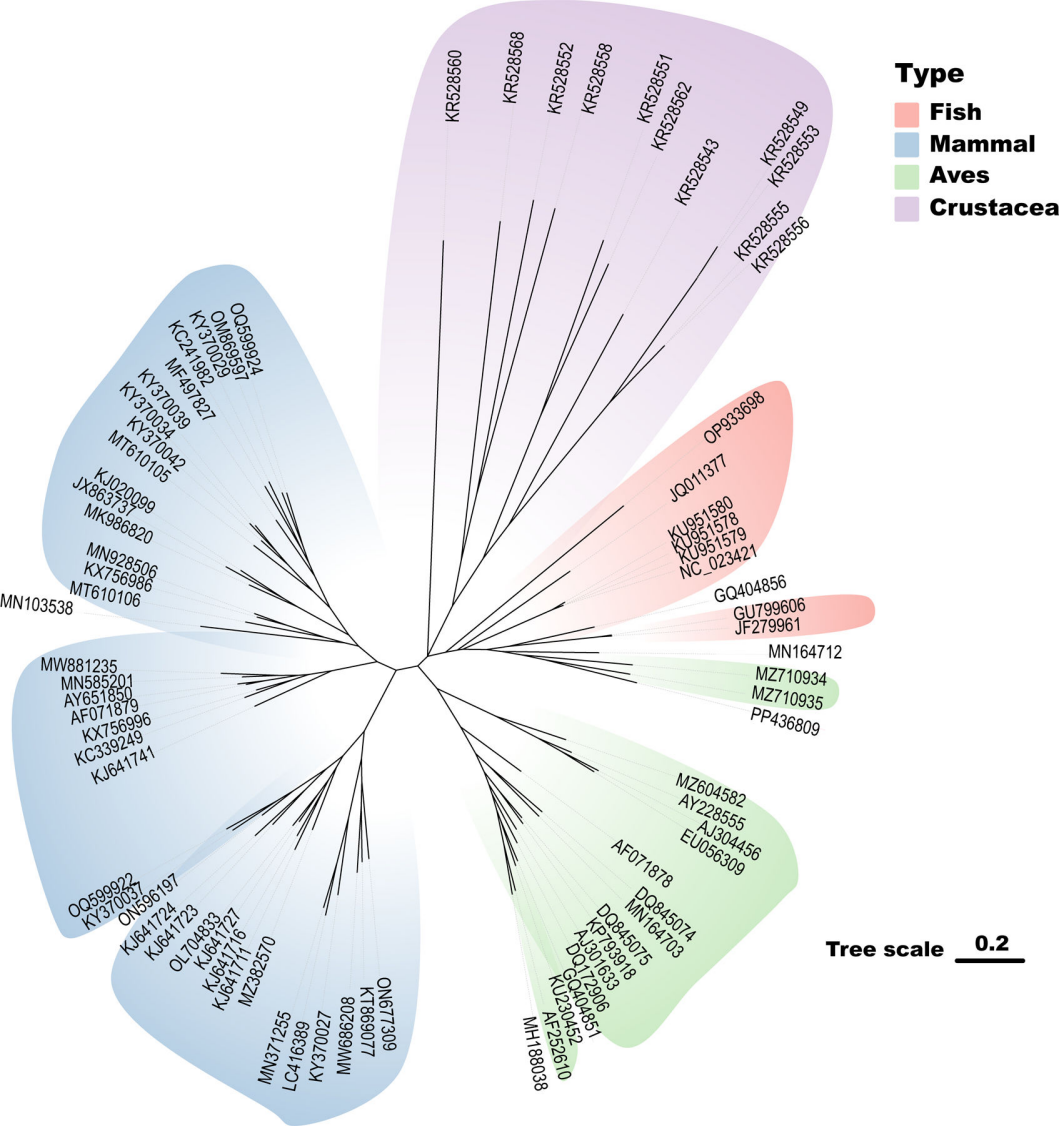
Phylogenetic analysis of circoviruses across various host types based on complete genome sequences. Maximum likelihood trees were constructed using MEGA-X (26) with 1,000 bootstrap replicates, employing the Tamura–Nei model to assess the tree’s topological stability. The sequences are categorized into four distinct clusters, with branches in each cluster color-coded for easy differentiation: Fish (Pink), Mammal (Blue), Aves (Green), and Crustacea (Purple). Fish circoviruses form a separate clade, suggesting host-specific evolution. Mammalian circoviruses cluster together, indicating a potential common ancestry. Avian circoviruses display a distinct grouping, reflecting their unique evolutionary path. Crustacean circoviruses form an outgroup, suggesting an early divergence from other lineages. Tree scale: 0.2 substitutions per site. Tree visualization by one table (tvBOT) (27).

Phylogenetic analysis of aquatic circoviruses revealed a complex evolutionary history, characterized by host-specific clustering and potential cross-species transmission events. Figure 3 presents a comprehensive phylogenetic tree based on whole- genome sequences, illustrating distinct clustering patterns among circoviruses from different aquatic hosts. The tree clearly separates circoviruses by host type, with aquatic circoviruses, especially those isolated from fish and crustaceans, predominantly grouped together. This clustering pattern suggests a closer evolutionary relationship among aquatic circoviruses compared with those from terrestrial hosts. Notably, crustacean-associated circoviruses form a separate cluster within the aquatic group but remain more closely related to fish circoviruses than to those from birds or mammals. This clustering pattern may reflect evolutionary relationships or similar adaptations driven by the aquatic environment.

**Fig 4:**
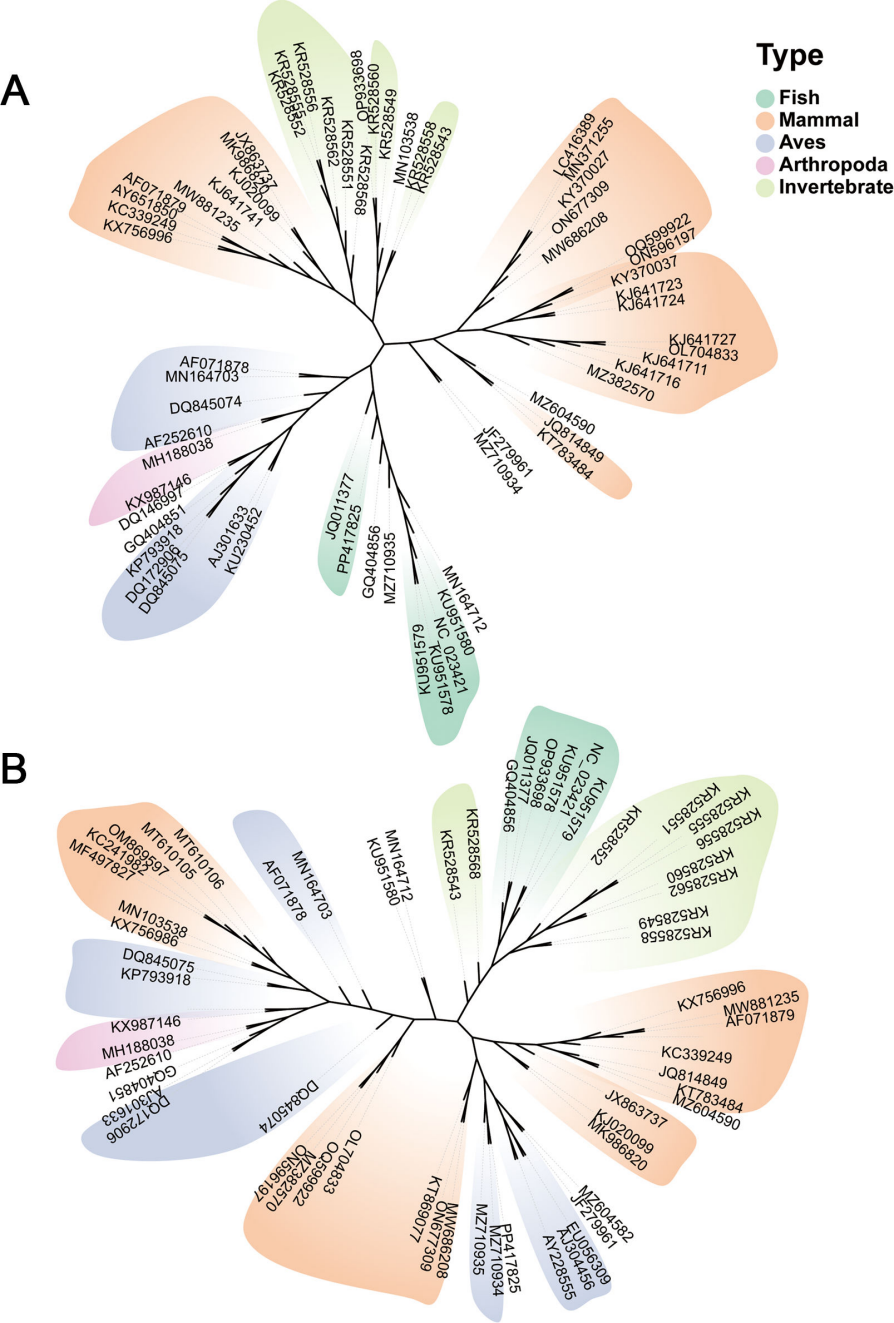
Phylogenetic analysis of circoviruses based on *Rep* and *CP* gene sequences. Maximum likelihood trees were generated using MEGA-X (26) with 1,000 bootstrap replicates, utilizing the LG + G model to evaluate the tree’s topological stability. (**A**) Phylogenetic tree based on *Rep* gene sequences. (**B**) Phylogenetic tree based on *CP* gene sequences. Tree visualization by one table (tvBOT) (27). The sequences are grouped into four distinct clusters, with branches in each cluster color-coded for clear differentiation.

**Fig 5:**
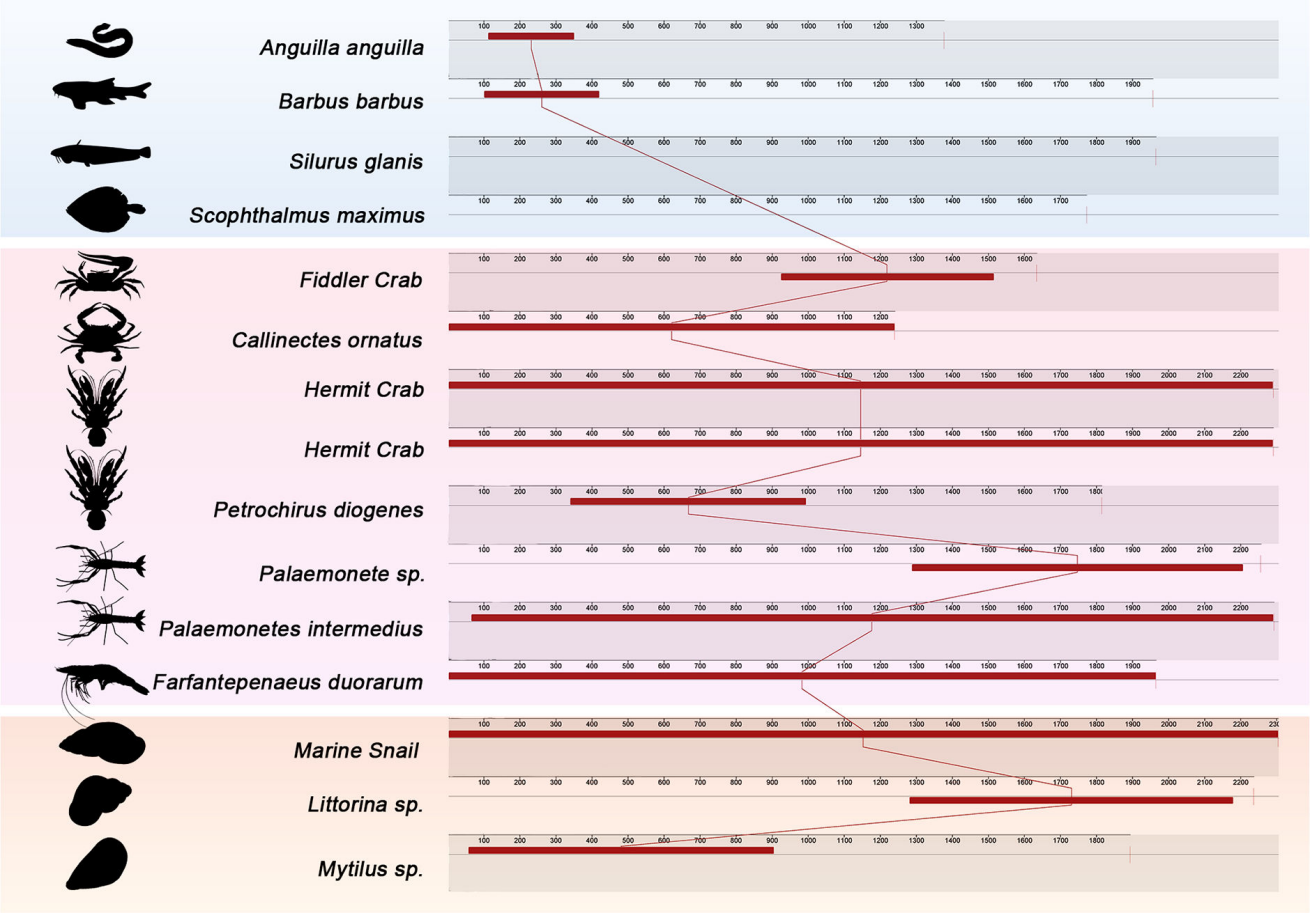
Mauve alignment of aquaculture-associated circovirus genomes. Colored blocks represent locally collinear blocks (LCBs) indicating conserved genomic regions across different circovirus isolates. The order and orientation of these blocks reflect gene organization, while gaps indicate regions of genomic rearrangement or variation. All organism silhouettes are from PhyloPic (www.phylopic.org).

Figure 4 shows separate phylogenetic analyses of the *Rep* and *CP* genes, respectively. These trees reveal some differences in evolutionary relationships compared to the whole-genome tree, suggesting potential recombination events or differential selective pressures on these genes. This observation aligns with reports of frequent recombination events in circoviruses, contributing to their genetic diversity and adaptability. The genome colinear analysis depicted in Fig. 5 further supports the diversity and evolutionary relationships among aquatic circoviruses. The colored blocks representing locally collinear regions highlight both conserved genomic elements and areas of significant variation across different circovirus isolates. This analysis reveals the dynamic nature of circovirus genomes, with evidence of gene rearrangements, insertions, and deletions that have occurred during their evolutionary history.

Circoviruses exhibit remarkable evolutionary dynamics, characterized by rapid evolution with substitution rates of approximately 1.2 × 10^−3^ per site per year (28). Their notable genetic diversity can be attributed to this elevated mutation rate in combination with frequent recombination events (29). While phylodynamic analyses have provided insights into the temporal evolution and population dynamics of circoviruses (30), our understanding of aquatic circoviruses specifically remains limited. The evolutionary trajectories of these viruses appear to be influenced by various environmental factors, including water temperature, host population density, and anthropogenic impacts. Furthermore, the discovery of integrated circovirus-like sequences in aquatic animal genomes (31) reveals complex host–pathogen relationships and suggests ancient virus–host associations. This evidence, along with potential cross-species transmission events, underscores the importance of further investigating the driving factors behind circovirus evolution and host range expansion.

### Geographical distribution and prevalence

**Fig 6:**
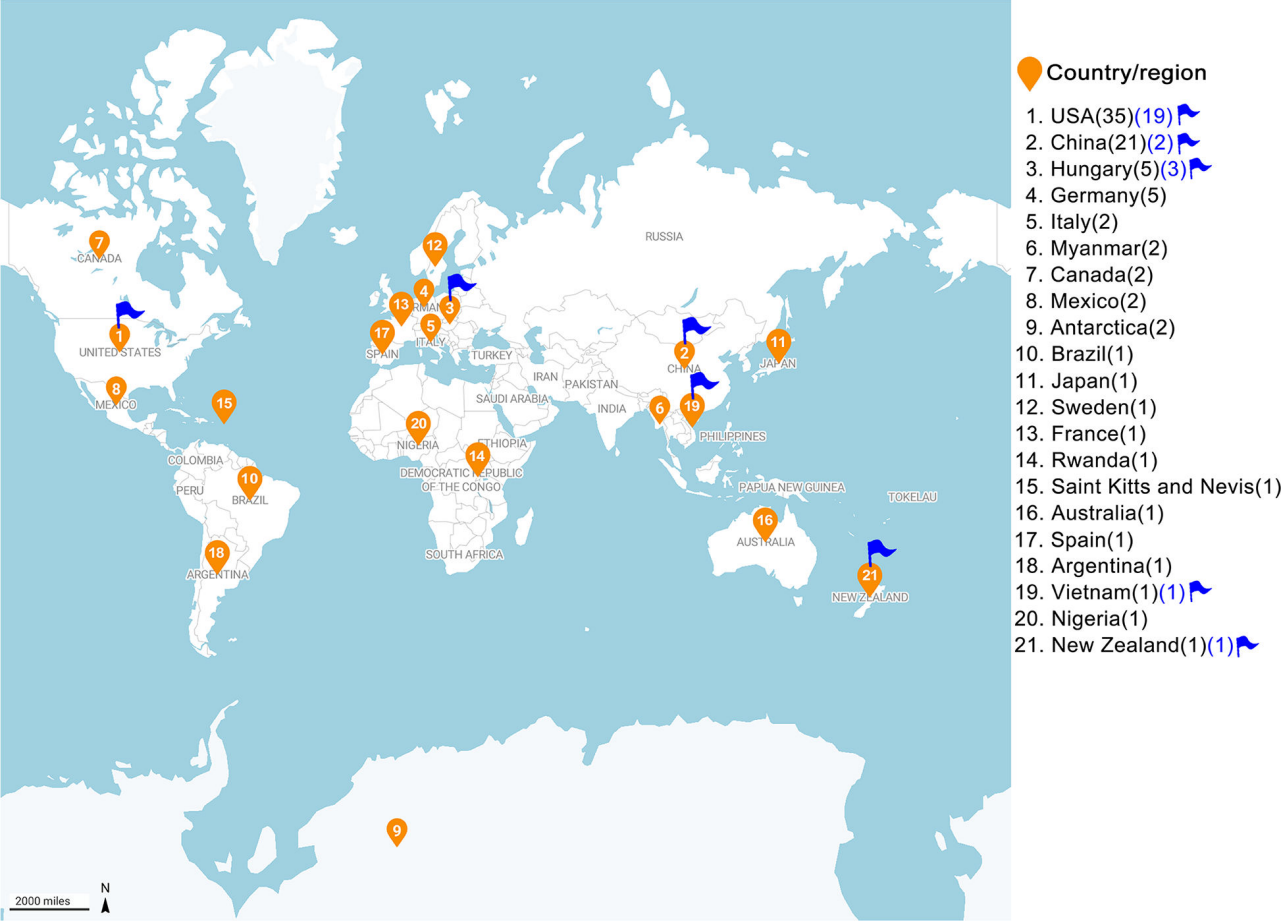
Global distribution of circoviruses with focus on aquatic species. Each numbered point represents a country or region, with the total number of circovirus species found there shown in parentheses. The blue numbers highlight the count of circovirus species specifically linked to aquatic animals in certain areas. This map was drawn using Datawrapper (https://www.datawrapper.de/).

Aquatic circoviruses demonstrate global distribution across all major continents, with significant diversity in both marine and freshwater environments. Current surveillance data indicate the highest documented diversity in the United States (19 aquatic-related species from 35 total circovirus species) and China (two aquatic species from 21 total species), with additional species identified in Hungary (three species) and Vietnam (one species) (Fig. 6). However, the true geographical distribution of these viruses is likely underestimated due to limited surveillance.

Regional surveillance has revealed distinct geographic patterns in circovirus distribution and diversity. In Asia, particularly China, intensive surveillance has yielded significant discoveries in aquaculture system, including TurCV in farmed *Scophthalmus maximus* populations along China’s northern coastline (7) and novel circoviruses in Hong Kong oysters (*Crassostrea hongkongensis*) from southern China (15). Additional findings encompass diverse viral populations in various crustacean species (9). North American surveillance has documented circovirus-like sequences in marine invertebrates from the Gulf of Mexico (9) and detected multiple circovirus variants in both farmed and wild fish populations (5, 6).

The identification of endogenous circoviral elements (CVEs) in globally distributed fish species suggests historical circovirus–host interactions across broad geographic ranges (31, 32). These distribution patterns appear influenced by multiple factors, including international aquaculture trade, migratory species movements, environmental persistence, and host species distribution. However, current geographic data likely underestimate true circovirus distribution due to several limitations: restricted systematic surveillance, sampling biases toward major aquaculture regions, variable detection and reporting capabilities, and focus on economically significant species. These constraints emphasize the critical need for comprehensive global surveillance programs, particularly in regions with emerging aquaculture industries, to establish a more complete understanding of aquatic circovirus distribution and prevalence patterns.

## CLINICAL SIGNS AND PATHOLOGICAL MANIFESTATIONS

### Single infection

Circovirus pathogenicity in aquatic species demonstrates substantial heterogeneity, ranging from asymptomatic infections to severe disease manifestations, with clinical presentations varying according to host species, viral strain characteristics, and environmental parameters. Experimental infections with TurCV have provided the most comprehensive data on its pathogenicity, revealing a cumulative mortality of 90%–95% within 21 days, with clinical signs appearing 4–6 days post-exposure (7). Affected fish exhibit clinical manifestations, such as ocular, mandibular, and anterior abdominal hemorrhages, fin base lesions, and abnormal swimming patterns characterized by spiral movements and vertical positioning. Histopathological examination reveals significant damage to cardiac tissue, splenic lesions, and renal pathology (7). Laboratory studies with juvenile turbot (50 g) at 14.5°C ± 1°C have corroborated these findings, showing an approximately 90% mortality rate (7); however, validation is needed. The pathogenic impact of TurCV on crustacean and mollusk species is less well understood. Viral sequences have been detected in both healthy and diseased samples, but there is no clear correlation between viral presence and disease state.

### Pathogen coinfections and disease complexity

Aquatic circovirus infections frequently occur in conjunction with other viral pathogens, complicating diagnostics and clinical manifestations (13). For instance, the concurrent detection of eel herpesvirus and eel circovirus in aquaculture presents challenges differential diagnosis based on gross pathology (33, 34). Coinfection patterns have been documented for various viral combinations, including nodavirus and eel herpesvirus, as well as multiple circovirus genotype. The pathogenic synergy between circoviruses and secondary pathogens may involve viral-induced immunomodulation, enhanced host susceptibility to opportunistic infections, and exacerbated disease severity due to concurrent pathogen. This complex disease ecology parallels observations in terrestrial circovirus infections, particularly porcine circovirus-associated disease (PCVAD), where cofactors play a significant role in disease progression (35, 36). The necessity for cofactors in terrestrial systems hints at analogous mechanisms operating in aquatic environments, where environmental stressors and coinfections may influence circovirus pathogenicity.

### Pathogenic mechanisms

The pathogenic mechanisms of aquatic circoviruses are gradually being elucidated through observational studies and emerging experimental evidence. Detailed histopathological analyses of TurCV-infected *S. maximus* have revealed comprehensive tissue alterations across multiple organ systems. In gill tissue, primary pathological changes include cellular infiltration and lymphocyte accumulation. The splenic tissue exhibits characteristic necrotic cord lesions, accompanied by splenocyte degeneration, nuclear deterioration, and cytoplasmic modifications including hypertrophy and vacuolation. Renal tissue demonstrates distinct hematopoietic cell modifications, chromatin marginalization, and nuclear fission patterns (7). Additionally, hepatic tissue shows pathological changes including hepatocyte swelling, vacuolar degeneration, and focal necrosis.

While the specific molecular mechanisms of aquatic circovirus pathogenicity remain under investigation, research insights from other circovirus systems suggest potential pathogenic pathways that warrant exploration in aquatic species. These may include cellular apoptosis mechanisms (37) and immune response modulation (38), though direct evidence in aquatic circoviruses is still limited. The distinct host–pathogen interactions in aquatic environments and the unique physiological characteristics of aquatic hosts necessitate focused research to elucidate the specific pathogenic mechanisms of aquatic circoviruses.

### Host–pathogen interactions and environmental modulation

Host–circovirus interactions in aquatic systems demonstrate complex multifactorial dependencies influenced by host physiology, viral characteristics, and environmental parameters. Key host determinants include age-dependent susceptibility, with enhanced vulnerability observed in juveniles possessing immature immune systems, as well as physiological status factors, such as nutritional condition and genetic background, that significantly affect disease outcomes. The host immunological response encompasses variable antibody production and cell-mediated responses across species. Environmental modulators, including temperature effects on viral transmission and pathogenicity, water quality parameters (salinity, pollutants), also impact the transmission and pathogenicity of aquatic circoviruses.

Host-specific transmission patterns are particularly evident in bivalve species, where filter-feeding behavior facilitates viral concentration, and population density affects transmission rates, establishing these organisms as significant viral repositories in marine ecosystems. Jiang et al*.* (15) proposed that oysters, owing to their filter-feeding lifestyle, ability to concentrate suspended microbes from the water column, and high population density, could serve as “repositories and transmission hotspots” for marine viruses (15). The presence of diverse viruses in filter-feeding shellfish has implications for both health of aquaculture and the viral ecology of coastal ecosystems. Recent research has advanced our understanding of circovirus-associated pathology and clinical manifestations in aquatic species, but significant knowledge gaps still exist. The observed variations in host range and pathogenic potential indicate the need for additional research to better understand the complex interplay between circoviruses, their aquatic hosts, and environmental factors.

## VIRUS ISOLATION AND CULTIVATION

Current limitations in aquatic circovirus research stem primarily from the absence of reliable cell culture systems, contrasting with established methods for terrestrial circoviruses such as PCV2 propagation in PK-15 cells (39). Unlike other viral pathogens affecting aquaculture species, the isolation and cultivation of aquatic circoviruses have proven particularly challenging (11). Initial isolation attempts have demonstrated limited success, as evidenced by the European eel circovirus, which showed promising initial cytopathic effects in EK-1 cells but failed to propagate in subsequent passages, with PCR confirmation remaining negative (11). Similarly, TurCV showed no obvious cytopathic effects in multiple fish cell lines, including carp papular epithelioma (EPC), salmon embryo (CHSE-214), and bluegill juvenile (BF-2) cell lines (7). However, emerging approaches in viral cultivation offer new possibilities for advancing aquatic circovirus research. These include preliminary trials of 3D culture systems (40), optimization of environmental parameters within a temperature range of 15°C–25°C, and systematic evaluation of various media supplement combinations. Furthermore, innovative genetic engineering approaches, particularly CRISPR-Cas9 technology (41, 42), have shown promise in terrestrial virus research and may be adapted for aquatic applications. These cutting-edge tools could facilitate the development of specialized aquatic cell lines optimized for viral replication, potentially breaking through the current cultivation barriers in aquatic circovirus research.

## DISEASE MANAGEMENT AND PREVENTION

### Diagnostic and detection methods

The detection and diagnosis of aquatic circoviruses have predominantly relied on nucleic acid amplification techniques, with PCR-based methodologies serving as the primary diagnostic tool. The development of a consensus-nested PCR system targeting conserved regions within the *Rep* gene has proven particularly effective for detecting diverse circovirus sequences (43). The diagnostic process typically begins with careful sample preparation, utilizing approximately 0.1 g of tissue from various organs, including the liver, spleen, gills, kidneys, gonads, intestine, brain, and blood. These samples undergo homogenization and clarification through low-speed centrifugation before DNA extraction using commercial kits (5, 6, 11, 44, 45). To enhance detection sensitivity, researchers have implemented advanced techniques such as multiple primed rolling circle amplification using exonuclease-resistant random primers for genomic copy pre-amplification prior to PCR analysis (46). The effectiveness of these methods has been demonstrated in recent surveillance studies, notably in the detection of TurCV, where researchers achieved an 80% positive detection rate in samples collected from multiple Chinese provinces between 2020 and 2023. This study revealed important tissue tropism patterns, with consistent detection in heart, spleen, and kidney tissues (7). Furthermore, species-specific applications have shown promising results, as exemplified by the development of a highly specific and sensitive PCR detection method for Eel circovirus (EeCV), which achieved a 40.6% positive detection rate in suspected viral disease samples (33).

These diagnostic advances have significantly improved our ability to detect and monitor aquatic circoviruses, contributing to better disease surveillance and management in aquaculture settings. The continued refinement of these techniques, coupled with their demonstrated reliability and specificity, has established PCR-based methods as the gold standard for aquatic circovirus detection and diagnosis.

### Vaccines and potential antiviral strategies

The development of vaccines and antiviral strategies for aquatic circoviruses has made significant progress, particularly with breakthroughs in TurCV research. While no commercial vaccines were initially available for aquatic circoviruses, recent advances have demonstrated promising results with subunit vaccines. Research has shown that recombinant capsid protein (rCap)-based vaccines, administered through both injection and oral routes, can provide significant protection in turbot. Intraperitoneal injection of rCap protein (1 mg/dose) achieved a 94.17% protection rate, while oral administration (5 mg/dose with a booster at 2 weeks) provided 88.33% protection against viral challenge (47). These findings represent a crucial advancement in aquatic circovirus vaccine development, establishing effective dosage parameters and administration routes for practical aquaculture applications.

Parallel developments in antiviral strategies have focused on immunological interventions and therapeutic approaches. Notable success has been achieved with antibody-based treatments, where monoclonal antibodies demonstrated 80% survival rates and polyclonal antibodies showed 65% survival rates in infected turbot when administered 3 days post-infection (47). Additional antiviral approaches under investigation include RNA interference (RNAi) technology targeting specific viral genes, broad-spectrum antivirals, and immunomodulators to enhance host innate immune responses. These advances collectively provide a comprehensive framework for both preventive and therapeutic strategies against aquatic circovirus infections, though challenges remain in optimizing delivery methods and maintaining efficacy in diverse aquaculture environments.

### Management strategies

Aquaculture currently employs various strategies for circovirus management, encompassing biosecurity measures like UV water treatment and filtration (with efficacy varying across applications), continual refinement of quarantine protocols, and assessment of standard sanitation practices for equipment. Monitoring effects entail regular sampling frequencies, ranging from weekly to monthly, using PCR-based detection methods. Key strategies include implementing strict quarantine protocols, screening broodstock for infections, and optimizing water quality and husbandry practices to minimize the risk of virus introduction and spread in aquaculture facilities. Regular monitoring using sensitive diagnostic tools enables early detection and rapid implementation of control measures, such as isolating or culling infected animals.

Vaccination is being investigated as a potential strategy to manage circovirus infections, although efficacy data in certain host species are limited, inactivated vaccines based on the capsid protein have shown varying levels of efficacy in fish (47). However, the rapid evolution and genetic diversity of circoviruses may complicate vaccine development and effectiveness. Selective breeding for disease resistance is another potential strategy, with genetic variation in host susceptibility observed in some fish and crustacean species. Marker-assisted breeding or genome editing could enhance the resilience of aquaculture populations, although the genetic basis for resistance in aquatic animals is still not well understood. While evidence for the effectiveness of these management strategies remains preliminary, systematic evaluation is necessary to establish standardized protocols and identify best practices.

## FUTURE DIRECTIONS AND CONCLUSIONS

Aquatic circoviruses are increasingly being recognized as emerging pathogens of concern in global aquaculture, with evidence suggesting their economic impacts and possible implications for the sustainability of fish and shellfish production systems. While recent advances in molecular biology and metagenomics have significantly expanded our understanding of their genetic diversity, host range, and ecological impacts, several critical knowledge gaps persist: (i) Pathogenicity and host impact: The pathogenic potential of virulence mechanisms of newly identified aquatic circoviruses remain incompletely characterized. (ii) Host range and transmission dynamics: Despite detection across diverse aquatic species, the full extent of host specificity and mechanisms driving interspecies transmission remain to be elucidated. (iii) Ecological significance: The role of circoviruses in aquatic ecosystem function, particularly in virus–host interactions and microbial community dynamics, necessitates further comprehensive investigation. (iv) Pathogen interactions: Detailed examination is required to understand the potential synergistic effects between circoviruses and other pathogens or environmental stressors in aquaculture systems. (v) Therapeutic interventions: As the importance of circoviruses in aquaculture grows, research into potential control measures, including vaccination strategies, becomes increasingly crucial.

Future research should prioritize elucidating molecular mechanisms of viral replication and pathogenesis, while also examining the impact of coinfections and environmental parameters on disease manifestation. Establishment of robust cell culture systems is a critical prerequisite for advancing our understanding of viral biology and accelerating therapeutic development. Several innovative technological approaches deserve implementation: (i) Integration of single-cell RNA sequencing with genome-wide CRISPR screening (10^4^–10^5^ targets) to delineate viral entry mechanisms, employing guide RNA libraries targeting predicted host receptors and restriction factors, followed by validation through comprehensive protein–protein interaction analyses (co-immunoprecipitation, bimolecular fluorescence complementation). This approach addresses the fundamental challenge of identifying susceptible cell types and determining essential host factors for viral infection, providing unprecedented resolution of virus–host interactions at the single-cell level. (ii) Implementation of nanopore sequencing with optimized circular DNA enrichment protocols (>95% purity), incorporating real-time variant calling (Q-score >12) and automated bioinformatics pipelines for outbreak surveillance, maintaining minimum 30× coverage for reliable variant detection. This method enables rapid, real-time tracking of circovirus variants during disease outbreaks and overcomes the technical challenges of sequencing circular viral genomes, facilitating immediate response to emerging threats. (iii) Development of multi-lineage aquatic tissue organoids incorporating physiologically relevant flow conditions (0.1–1.0 dyne/cm^2^), temperature regulation (15°C–25°C), and tissue-specific extracellular matrices, validated through transcriptome comparison with primary tissues (>80% similarity threshold). This system provides a solution to studying circovirus infections under conditions that closely mimic the natural aquatic environment, enabling investigation of how environmental parameters influence viral pathogenesis in different tissue types. (iv) Application of deep learning models trained on comprehensive viral genomic data sets (*n* > 1000 sequences) and host infection profiles, utilizing attention-based architectures for prediction of tissue tropism (AUC >0.85) and host range expansion, with experimental validation of predicted interactions. This computational approach enables prediction of potential host range expansion and tissue tropism before outbreaks occur, allowing proactive implementation of preventive measures. (v) Implementation of CRISPR-Cas9 approaches targeting conserved viral replication elements and host factors, utilizing multiplexed guide RNAs (efficiency >80%) and inducible systems for temporal control, verified through deep sequencing of edited sites (indel frequency >90%). This technique provides a powerful tool for validating essential viral and host factors while offering potential therapeutic strategies through targeted genetic modification. These methodological approaches require standardized protocols, rigorous controls, and integration with existing aquaculture biosecurity frameworks, while maintaining practical feasibility for industry implementation. The combined application of these techniques addresses the major challenges in understanding aquatic circovirus biology, from molecular mechanisms to practical disease management. Success metrics encompass reproducibility across host species, validation under field conditions, and demonstrated improvement in disease management outcomes, ultimately advancing both our fundamental knowledge and practical control strategies for aquatic circoviruses.

In conclusion, aquatic circoviruses represent an emerging challenge to sustainable aquaculture development, necessitating coordinated efforts among virologists, veterinarians, and aquaculture specialists. Through the integration of advanced scientific methodologies and fostering of interdisciplinary collaboration, the scientific community can work toward mitigating the impact of these pathogens and enhancing the resilience of aquaculture production systems.

## SUPPLEMENTAL MATERIAL

Fig. S1 jvi.01737-24-s0001.docx Amino acid sequence alignment of multiple CP proteins from various aquatic circoviruses.

Table S1 jvi.01737-24-s0002.docx Summary of representative members of the genus *Circovirus* and aquatic-associated circoviruses.

ASM does not own the copyrights to Supplemental Material that may be linked to, or accessed through, an article. The authors have granted ASM a non-exclusive, world-wide license to publish the Supplemental Material files. Please contact the corresponding author directly for reuse.
