# Supplementary material for: Aquatic circoviruses: emerging pathogens in global aquaculture — from discovery to disease management
Source: J Virol. 2024 Dec 13;99(1):e01737-24. doi: 10.1128/jvi.01737-24 (PMC11784310; doi:10.1128/jvi.01737-24)
Supplement: Fig. S1 — Amino acid sequence alignment of multiple CP proteins from various aquatic circoviruses. [file jvi.01737-24-s0001.docx]

**Supplementary Figure S1**


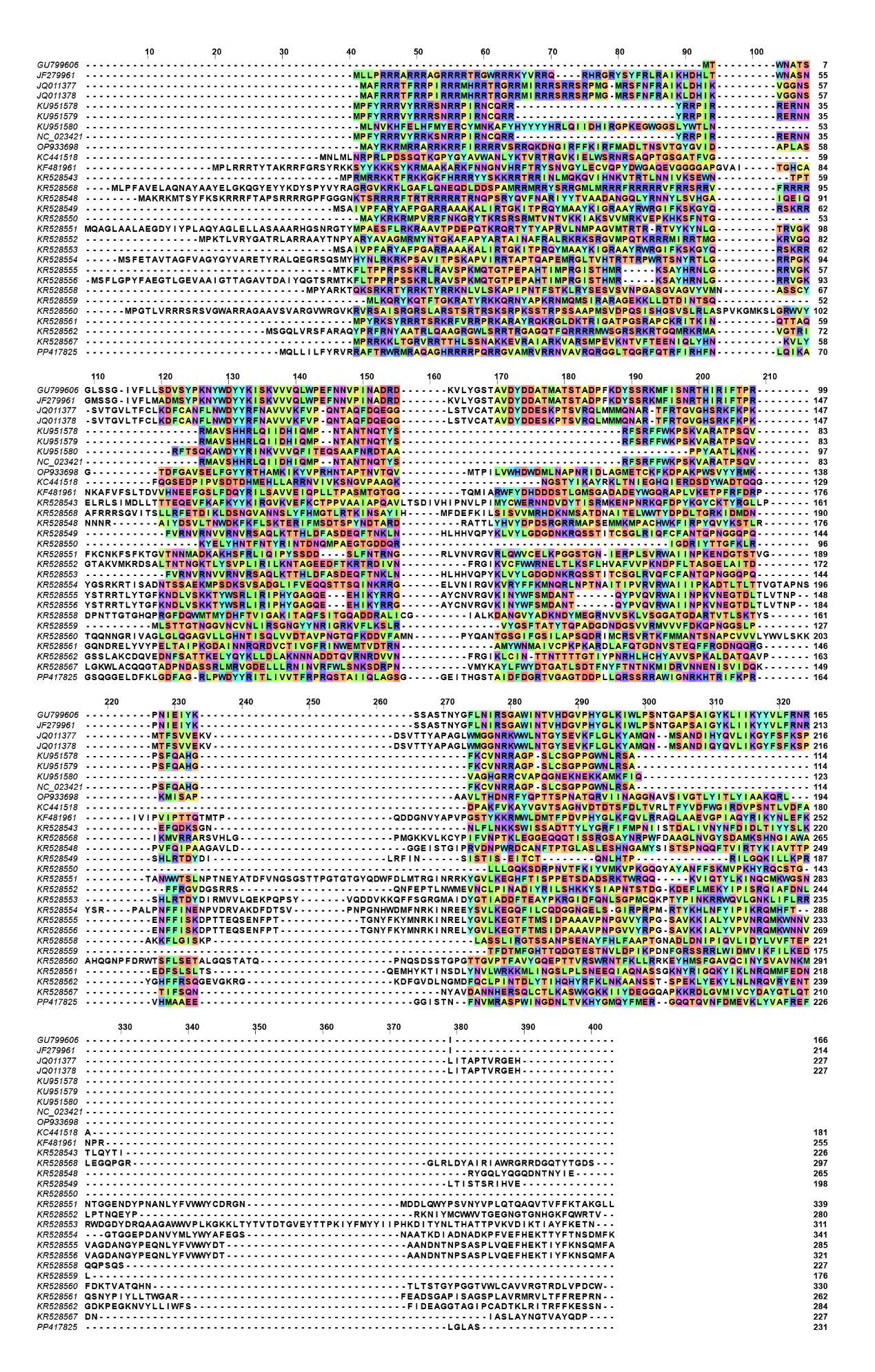


**Figure S1** Amino acid sequence alignment of multiple CP proteins from various aquatic circoviruses. The alignment is visualized using Jalview software (72). Each row represents a different virus strain, and the columns represent the amino acid positions. Different colors are used to highlight conserved and variable regions across the sequences.
