# Supplementary material for: Aquatic circoviruses: emerging pathogens in global aquaculture — from discovery to disease management
Source: J Virol. 2024 Dec 13;99(1):e01737-24. doi: 10.1128/jvi.01737-24 (PMC11784310; doi:10.1128/jvi.01737-24)
Supplement: Table S1 — Summary of representative members of the genus Circovirus and aquatic-associated circoviruses. [file jvi.01737-24-s0002.docx]

**Table S1** Summary of representative members of the genus *Circovirus* and aquatic-associated circoviruses.

| **Binomial**  **species name** | **Epithet notes** | **Previous species**  **name** | **Accession no.** | **Virus name** | **Host /source** | **Country** | **Ref** |
| --- | --- | --- | --- | --- | --- | --- | --- |
| Circovirus ban | coyote in Tohono O’odham | - | OQ599924 | banfec circovirus 2 | *Canis latrans* | USA | (17) |
| Circovirus barbel | common name for host | Barbel circovirus | GU799606 | barbel circovirus | *Barbus barbus* | Hungary | (3) |
| Circovirus bastao | bat in Portuguese | Bat associated circovirus 4 | KT783484 | Tadarida brasiliensis circovirus 1 | *Tadarida brasiliensis* | Brazil | (18) |
| Circovirus bear | common name for host | Bear circovirus | MN371255 | Ursus americanus circovirus | *Ursus americanus americanus* | USA | (19) |
| Circovirus bianfu | bat in Chinese | Bat associated circovirus 3 | JQ814849 | Rhinolophus ferrumequinum circovirus 1 | *Rhinolophus ferrumequinum* | China | (20) |
| Circovirus canary | common name for host | Canary circovirus | AJ301633 | canary circovirus | *Serinus canaria* | Italy | (21) |
| Circovirus canine | common name for host | Canine circovirus | KC241982 | canine circovirus | *Canis lupus familiaris* | USA | (22) |
| Circovirus catfish | common name for host | European catfish circovirus | JQ011377 | Silurus glanis circovirus | *Silurus glanis* | Hungary | (4) |
| Circovirus chauvesouris | bat in French (2 words) | Bat associated circovirus 1 | JX863737 | bat associated circovirus 1 | *Rhinolophus ferrumequinum* | Myanmar | (23) |
| Circovirus cia | bat in Lao | Bat associated circovirus 12 | KJ641716 | bat circovirus CV/GD2012 | *Pipistrellus sp.* | China | (24) |
| Circovirus civet | common name for host | Civet circovirus | LC416389 | Paguma larvata circovirus | *Paguma larvata* | Japan | (25) |
| Circovirus daga | rodent in Filipino | Rodent associated circovirus 6 | KY370037 | rodent circovirus 6 | *Apodemus draco* | China | (26) |
| Circovirus duck | common name for host | Duck circovirus | AY228555 | mulard duck circovirus | *Anas domesticus* | USA | (27) |
| Circovirus elk | common name for host | Elk circovirus | MN585201 | elk circovirus | *Cervus canadensis* | Canada | (28) |
| Circovirus eniyan | human in Yoruba | Human associated circovirus 1 | GQ404856 | human stool associated circular virus | *Homo sapiens* | Nigeria | (29) |
| Circovirus finch | common name for host | Finch circovirus | DQ845075 | finch circovirus | *Chloebia gouldiae* | - | (30) |
| Circovirus gloton | wolverine in Spanish | - | MW686208 | wolvfec circovirus | *Gulo gulo* | USA | (31) |
| Circovirus gnaver | rodent in Danish | Rodent associated circovirus 5 | KY370027 | rodent circovirus 5 | *Niviventer eha* | China | (26) |
| Circovirus goose | common name for host | Goose circovirus | AJ304456 | goose circovirus | *Goose* | Germany | (32) |
| Circovirus gryzon | rodent in Polish | Rodent associated circovirus 7 | MF497827 | bamboo rat circovirus | *Rhizomys pruinosus* | China | - |
| Circovirus gull | common name for host | Gull circovirus | DQ845074 | *gull circovirus* | *Larus argentatus* | Sweden | (30) |
| Circovirus gyurgyalag | bee-eater in Hungarian | - | MZ710935 | bee-eater circovirus | *Merops apiaster* | Hungary | (33) |
| Circovirus hirat | rat in Volapuk | - | OM869597 | dipodfec virus UA04Rod_4537 | *Dipodomys merriami feces* | USA | (34) |
| Circovirus human | virus identified in human tissue | - | ON677309 | human circovirus 1 | *Homo sapiens* | France | (35) |
| Circovirus ialtag | bat in Scottish | Bat associated circovirus 10 | KX756986 | bat circovirus Acheng30 | *Vespertilio sinensis* | China | (36) |
| Circovirus impundu | chimpanzee in Kinyarwanda | Chimpanzee associated circovirus 1 | GQ404851 | chimpanzee stool avian-like circovirus | *Pan troglodytes* | Rwanda | (29) |
| Circovirus kelawar | bat in Malay | Bat associated circovirus 9 | KJ641741 | bat associated circovirus 9 | *Rhinolophus ferrumequinum* | China | (24) |
| Circovirus kiore | rodent in Māori | Rodent associated circovirus 2 | KY370042 | rodent circovirus 2 | *Apodemus chevrieri* | China | (26) |
| Circovirus kukwuria | coyote, scavenger in Comanche | - | OQ599922 | banfec circovirus 1 | *Canis latrans* | USA | (17) |
| Circovirus lepakko | bat in Finnish | Bat associated circovirus 7 | KJ641723 | bat associated circovirus 7 | *Rhinolophus sinicus* | China | (24) |
| Circovirus lin | horse in Navajo | - | MW881235 | equine circovirus 1 | *Equus ferus caballus* | USA | (37) |
| Circovirus magu | mongoose in Punjabi | - | MZ382570 | mongoose-associated circovirus Mon-1 | *Urva auropunctata* | Saint Kitts and Nevis | (38) |
| Circovirus mink | common name for host | Mink circovirus | KJ020099 | mink circovirus | *Mustela sp.* | China | (39) |
| Circovirus miztli | mountain lion in Nahuatl | - | MT610105 | sonfela circovirus 1 | *Lynx rufus* | Mexico | (40) |
| Circovirus miztontli | cat in Milpa Alta | - | MT610106 | sonfela circovirus 2 | *Lynx rufus* | Mexico | (40) |
| Circovirus morcego | bat in Galician | Bat associated circovirus 8 | KJ641711 | bat associated circovirus 8 | *Myotis ricketti* | China | (24) |
| Circovirus mossi | common word for mosquito in USA | Mosquito associated circovirus 1 | MH188038 | Culex circovirus- like virus | *Culex sp.* | USA | (41) |
| Circovirus naaleeli | waterfowl in Navajo | - | MZ604582 | wigfec circovirus 1 | *Mareca americana* | USA | (42) |
| Circovirus parrot | common name for host | Beak and feather disease virus | AF071878 | beak and feather disease virus | *Psittaciformes species* | USA | (43) |
| Circovirus pato | waterfowl in Spanish | - | MZ604590 | wigfec circovirus 2 | *Mareca americana* | USA | (42) |
| Circovirus penguin | common name for host | Penguin circovirus | MN164703 | penguin circovirus | *Pygoscelis adeliae* | Antarctica | (44) |
| Circovirus pichong | tick in Chinese (two words Pí chóng) | Tick associated circovirus 2 | KX987146 | tick circovirus 2 | *Ixodes crenulatus* | China | (45) |
| Circovirus pigeon | common name for host | Pigeon circovirus | AF252610 | columbid circovirus | *Columbia livia* | Germany | (46) |
| Circovirus pipistrello | bat in Italian | Bat associated circovirus 13 | MN928506 | bat circovirus Sardinia | *Miniopterus schreibersii* | Italy | (47) |
| Circovirus porcine1 | common name for host | Porcine circovirus 1 | AF071879 | porcine circovirus 1 | *Sus scrofa domes- ticus* | Germany | (43) |
| Circovirus porcine2 | common name for host | Porcine circovirus 2 | AY651850 | porcine circovirus 2 | *Sus scrofa domes- ticus* | Canada | (48) |
| Circovirus porcine3 | common name for host | Porcine circovirus 3 | KT869077 | porcine circovirus 3 | *Sus scrofa domes- ticus* | USA | (49) |
| Circovirus porcine4 | common name for host | Porcine circovirus 4 | MK986820 | porcine circovirus 4 | *Sus scrofa domes- ticus* | China | (50) |
| Circovirus ratpenat | bat in Catalan | Bat associated circovirus 6 | KJ641724 | bat associated circovirus 6 | *Rhinolophus affinis* | China | (24) |
| Circovirus raven | common name for host | Raven circovirus | DQ146997 | raven circovirus | *Corvus coronoides* | Australia | (51) |
| Circovirus roditore | rodent in Italian | Rodent associated circovirus 3 | KY370039 | rodent circovirus 3 | *Neodon clarkei* | China | (26) |
| Circovirus rongeur | rodent in French | Rodent associated circovirus 1 | KY370034 | rodent circovirus 1 | *Neodon clarkei* | China | (26) |
| Circovirus rosegador | rodent in Catalan | Rodent associated circovirus 4 | KY370029 | rodent circovirus 4 | *Allactaga sibirica* | China | (26) |
| Circovirus saguzarra | bat in Basque | Bat associated circovirus 5 | KJ641727 | bat associated circovirus 5 | *Plecotus auritus* | China | (24) |
| Circovirus siksparnis | bat in Latvian | Bat associated circovirus 11 | KX756996 | bat circovirus Mengyuan2 | *Hipposideros armiger* | China | (26) |
| Circovirus starling | common name for host | Starling circovirus | DQ172906 | starling circovirus | *Sturnus vulgaris* | Spain | (52) |
| Circovirus swan | common name for host | Swan circovirus | EU056309 | Cygnus olor circovirus | *Cygnus olor* | Germany | (53) |
| Circovirus tetning | seal in Norwegian | - | MN164712 | werosea circovirus | *Leptonychotes weddellii* | Antarctica | (54) |
| Circovirus torpegem | little bittern in Hungarian | - | MZ710934 | little bittern circovirus | *Ixobrychus minutus* | Hungary | (33) |
| Circovirus tzinaka | bat in Nahuatl | - | OL704833 | Eumops bonariensis associated circovirus 1 | *Eumops bonariensis* | Argentina | (55) |
| Circovirus vleermuis | bat in Dutch | Bat associated circovirus 2 | KC339249 | bat associated circovirus 2 | *Rhinolophus ferrume-quinum* | Myanmar | (23) |
| Circovirus wesa | cat in Cherokee | - | ON596197 | calfel virus LSF45 cir359 | *Lynx rufus* | USA | (84) |
| Circovirus whale | common name for host | Whale circovirus | MN103538 | beaked whale circovirus | *Indopacetus pacifcus* | USA | (57) |
| Circovirus yaa | tick in Navajo | Tick associated circovirus 1 | KU230452 | avian-like circovirus | *Ixodes scapulari* | USA | (58) |
| Circovirus zebrafinch | common name for host | Zebra finch circovirus | KP793918 | zebra finch circovirus | *Taeniopygia guttata* | Germany | (59) |
| Circovirus eel^1^ | common name for host | European eel circovirus | NC_023421 | Anguilla anguilla circovirus | *Anguilla anguilla* | Hungary | (9) |
| Circovirus Lhasa^1^ | - | Fish-associated circovirus | OP933698 | Fish-associated circovirus | *Fish in Lhasa River* | China | - |
| Circovirus Turbot^1^ | common name for host | Turbot circovirus | PP417825 | Scophthalmus maximus  circovirus | *Scophthalmus maximus* | China | (5) |
| Circovirus duorarum^1^ | common name for host | Farfantepenaeus duorarum circovirus | KC441518 | *Farfantepenaeus duorarum* circular  virus | *Farfantepenaeus duorarum* (hepatopancreas) | USA | (11) |
| Circovirus monodon^1^ | common name for host | Penaeus monodon circovirus VN11 | KF481961 | *Penaeus monodon* circovirus VN11 | *Penaeus monodon* | Vietnam | (60) |
| - | common name for host | Petrochirus diogenes giant hermit crab aCV^2^ | KR528543 | *P. diogenes* circular  virus | *Petrochirus diogenes*  (Abdomen) | USA | (7) |
| - | common name for host | *Palaemonete* sp. Common Grass Shrimp aCV^2^ | KR528568 | *Palaemonete* sp. circular  virus | *Palaemonete sp.*  (Hepatopancreas) | USA | (7) |
| - | common name for host | Littorina sp.Snail aCV^2^ | KR528548 | *Littorina* sp. circular  virus | *Littorina* sp. | USA | (7) |
| - | common name for host | C.ornatus Ornate Blue Crab aCV^2^ | KR528549 | Callinectes ornatus circular  virus | *Callinectes ornatus* (Gonads) | USA | (7) |
| - | common name for host | C.sapidus Atlantic Blue Crab aCV^2^ | KR528550 | Callinectes sapidus  circular  virus | *Callinectes sapidus* (Gonads) | USA | (7) |
| - | common name for host | *P. intermedius* Brackish Grass Shrimp aCV^2^ | KR528551 | Palaemonetes intermedius  circular  virus | *Palaemonetes intermedius* | USA | (7) |
| - | common name for host | *F.duorarum* Pink Shrimp aCV^2^ | KR528552 | Farfantepenaeus duorarum circular  virus | *Farfantepenaeus duorarum* | USA | (7) |
| - | common name for host | *F.duorarum* Pink Shrimp aCV^2^ | KR528553 | Farfantepenaeus duorarum circular  virus | *Farfantepenaeus duorarum* | USA | (7) |
| - | common name for host | Marine Snail aCV^2^ | KR528554 | Marine Snail circular  virus | *Marine Snail* | USA | (7) |
| - | common name for host | Hermit Crab aCV^2^ | KR528555 | Hermit Crab  circular  virus | *Hermit Crab* (Abdomen) | USA | (7) |
| - | common name for host | Hermit Crab aCV^2^ | KR528556 | Hermit Crab  circular  virus | *Hermit Crab* (Abdomen) | USA | (7) |
| - | common name for host | Hermit Crab aCG^2^ | KR528557 | Hermit Crab  circular  virus | *Hermit Crab* (Abdomen) | USA | (7) |
| - | common name for host | Fiddler Crab aCV^2^ | KR528558 | Fiddler Crab circular  virus | *Fiddler Crab* (Gonads and claw muscle) | USA | (7) |
| - | common name for host | Fiddler Crab aCV^2^ | KR528559 | Fiddler Crab circular  virus | *Fiddler Crab* (Gonads and claw muscle) | USA | (7) |
| - | common name for host | *P.kadiakensis* Mississippi Grass Shrimp aCV^2^ | KR528560 | *Palaemonetes kadiakensis* circular  virus | *Palaemonetes kadiakensis* | USA | (7) |
| - | common name for host | Gammarus sp. Amphipod aCV^2^ | KR528561 | Gammarus sp. circular  virus | *Gammarus sp.* | USA | (7) |
| - | common name for host | Mytilus sp. Clam aCV^2^ | KR528562 | Mytilus sp.  circular  virus | *Mytilus sp.* | USA | (7) |
| - | common name for host | Gastropod associated circular ssDNA virus | KC172652 | Gastropod-associated circular ssDNA virus (GaCSV) | *Amphibola*  *crenata* | New Zealand | (12) |
| - | common name for host | S. brevirostris Brown Rock Shrimp aCV^2^ | KR528567 | Sicyonia brevirostris circular  virus | *Sicyonia brevirostris*  (Gonads) | USA | (7) |

^1^ Binomial species name are proposed in this article based on the ICTV naming method for virus-related species (10).

^2^ Previous species names contain abbreviation aCV for associated circular virus or aCG for associated circular genome.

**REFERENCES**

17. Hess SC, Weiss K, Custer JM, Lewis JS, Kraberger S, Varsani A. 2023. Identification of small circular DNA viruses in coyote fecal samples from Arizona (USA). Arch Virol 169:12.

18. Lima FE, Cibulski SP, Dall BA, Mayer FQ, Witt AA, Roehe PM, D'Azevedo PA. 2015. A novel chiropteran circovirus genome recovered from a Brazilian insectivorous bat species. Genome Announc 3.

19. Alex CE, Fahsbender E, Altan E, Bildfell R, Wolff P, Jin L, Black W, Jackson K, Woods L, Munk B, Tse T, Delwart E, Pesavento PA. 2020. Viruses in unexplained encephalitis cases in American black bears (*Ursus americanus*). PLoS One 15:e0244056.

20. Wu Z, Ren X, Yang L, Hu Y, Yang J, He G, Zhang J, Dong J, Sun L, Du J, Liu L, Xue Y, Wang J, Yang F, Zhang S, Jin Q. 2012. Virome analysis for identification of novel mammalian viruses in bat species from Chinese provinces. J Virol 86:10999-11012.

21. Todd D, Weston J, Ball NW, Borghmans BJ, Smyth JA, Gelmini L, Lavazza A. 2001. Nucleotide sequence-based identification of a novel circovirus of canaries. Avian Pathol 30:321-325.

22. Li L, McGraw S, Zhu K, Leutenegger CM, Marks SL, Kubiski S, Gaffney P, Dela CFJ, Wang C, Delwart E, Pesavento PA. 2013. Circovirus in tissues of dogs with vasculitis and hemorrhage. Emerg Infect Dis 19:534-541.

23. He B, Li Z, Yang F, Zheng J, Feng Y, Guo H, Li Y, Wang Y, Su N, Zhang F, Fan Q, Tu C. 2013. Virome profiling of bats from Myanmar by metagenomic analysis of tissue samples reveals more novel Mammalian viruses. PLoS One 8:e61950.

24. Wu Z, Yang L, Ren X, He G, Zhang J, Yang J, Qian Z, Dong J, Sun L, Zhu Y, Du J, Yang F, Zhang S, Jin Q. 2016. Deciphering the bat virome catalog to better understand the ecological diversity of bat viruses and the bat origin of emerging infectious diseases. ISME J 10:609-620.

25. Nishizawa T, Sugimoto Y, Takeda T, Kodera Y, Hatano Y, Takahashi M, Okamoto H. 2018. Identification and full-genome characterization of novel circoviruses in masked palm civets (*Paguma larvata*). Virus Res 258:50-54.

26. Wu Z, Lu L, Du J, Yang L, Ren X, Liu B, Jiang J, Yang J, Dong J, Sun L, Zhu Y, Li Y, Zheng D, Zhang C, Su H, Zheng Y, Zhou H, Zhu G, Li H, Chmura A, Yang F, Daszak P, Wang J, Liu Q, Jin Q. 2018. Comparative analysis of rodent and small mammal viromes to better understand the wildlife origin of emerging infectious diseases. Microbiome 6:178.

27. Hattermann K, Schmitt C, Soike D, Mankertz A. 2003. Cloning and sequencing of Duck circovirus (DuCV). Arch Virol 148:2471-2480.

28. Fisher M, Harrison T, Nebroski M, Kruczkiewicz P, Rothenburger JL, Ambagala A, Macbeth B, Lung O. 2020. Discovery and comparative genomic analysis of elk circovirus (ElkCV), a novel circovirus species and the first reported from a cervid host. Sci Rep 10:19548.

29. Li L, Kapoor A, Slikas B, Bamidele OS, Wang C, Shaukat S, Masroor MA, Wilson ML, Ndjango JB, Peeters M, Gross-Camp ND, Muller MN, Hahn BH, Wolfe ND, Triki H, Bartkus J, Zaidi SZ, Delwart E. 2010. Multiple diverse circoviruses infect farm animals and are commonly found in human and chimpanzee feces. J Virol 84:1674-1682.

30. Todd D, Scott AN, Fringuelli E, Shivraprasad HL, Gavier-Widen D, Smyth JA. 2007. Molecular characterization of novel circoviruses from finch and gull. Avian Pathol 36:75-81.

31. Bandoo RA, Bautista J, Lund M, Newkirk E, Squires J, Varsani A, Kraberger S. 2021. Identification of novel circovirus and anelloviruses from wolverines using a non-invasive faecal sampling approach. Infect Genet Evol 93:104914.

32. Todd D, Weston JH, Soike D, Smyth JA. 2001. Genome sequence determinations and analyses of novel circoviruses from goose and pigeon. Virology 286:354-362.

33. Feher E, Kaszab E, Bali K, Hoitsy M, Sos E, Banyai K. 2022. Novel circoviruses from birds share common evolutionary roots with fish origin circoviruses. Life (Basel) 12.

34. Lund MC, Larsen BB, Rowsey DM, Otto HW, Gryseels S, Kraberger S, Custer JM, Steger L, Yule KM, Harris RE, Worobey M, Van Doorslaer K, Upham NS, Varsani A. 2023. Using archived and biocollection samples towards deciphering the DNA virus diversity associated with rodent species in the families cricetidae and heteromyidae. Virology 585:42-60.

35. Perot P, Fourgeaud J, Rouzaud C, Regnault B, Da RN, Fontaine H, Le Pavec J, Dolidon S, Garzaro M, Chretien D, Morcrette G, Molina TJ, Ferroni A, Leruez-Ville M, Lortholary O, Jamet A, Eloit M. 2023. Circovirus hepatitis infection in heart-lung transplant patient, France. Emerg Infect Dis 29:286-293.

36. Zhu A, Jiang T, Hu T, Mi S, Zhao Z, Zhang F, Feng J, Fan Q, He B, Tu C. 2018. Molecular characterization of a novel bat-associated circovirus with a poly-T tract in the 3' intergenic region. Virus Res 250:95-103.

37. Hui A, Altan E, Slovis N, Fletcher C, Deng X, Delwart E. 2021. Circovirus in blood of a febrile horse with hepatitis. Viruses 13.

38. Gainor K, Becker A, Malik YS, Ghosh S. 2021. Detection and complete genome analysis of *Circoviruses* and *Cycloviruses* in the Small Indian Mongoose (*Urva auropunctata*): Identification of novel species. Viruses 13.

39. Lian H, Liu Y, Li N, Wang Y, Zhang S, Hu R. 2014. Novel circovirus from mink, China. Emerg Infect Dis 20:1548-1550.

40. Payne N, Kraberger S, Fontenele RS, Schmidlin K, Bergeman MH, Cassaigne I, Culver M, Varsani A, Van Doorslaer K. 2020. Novel circoviruses detected in feces of sonoran felids. Viruses 12.

41. Sadeghi M, Altan E, Deng X, Barker CM, Fang Y, Coffey LL, Delwart E. 2018. Virome of > 12 thousand culex mosquitoes from throughout California. Virology 523:74-88.

42. Khalifeh A, Custer JM, Kraberger S, Varsani A. 2021. Novel viruses belonging to the family *Circoviridae* identified in wild American wigeon samples. Arch Virol 166:3437-3441.

43. Niagro FD, Forsthoefel AN, Lawther RP, Kamalanathan L, Ritchie BW, Latimer KS, Lukert PD. 1998. Beak and feather disease virus and porcine circovirus genomes: intermediates between the geminiviruses and plant circoviruses. Arch Virol 143:1723-1744.

44. Morandini V, Dugger KM, Ballard G, Elrod M, Schmidt A, Ruoppolo V, Lescroel A, Jongsomjit D, Massaro M, Pennycook J, Kooyman GL, Schmidlin K, Kraberger S, Ainley DG, Varsani A. 2019. Identification of a novel Adelie Penguin circovirus at Cape Crozier (Ross Island, Antarctica). Viruses 11.

45. Wang B, Sun LD, Liu HH, Wang ZD, Zhao YK, Wang W, Liu Q. 2018. Molecular detection of novel circoviruses in ticks in northeastern China. Ticks Tick Borne Dis 9:836-839.

46. Mankertz A, Hattermann K, Ehlers B, Soike D. 2000. Cloning and sequencing of *columbid circovirus* (coCV), a new circovirus from pigeons. Arch Virol 145:2469-2479.

47. Lecis R, Mucedda M, Pidinchedda E, Zobba R, Pittau M, Alberti A. 2020. Genomic characterization of a novel bat-associated Circovirus detected in European *Miniopterus schreibersii* bats. Virus Genes 56:325-328.

48. Hamel AL, Lin LL, Nayar GP. 1998. Nucleotide sequence of porcine circovirus associated with postweaning multisystemic wasting syndrome in pigs. J Virol 72:5262-5267.

49. Palinski R, Pineyro P, Shang P, Yuan F, Guo R, Fang Y, Byers E, Hause BM. 2017. A novel porcine circovirus distantly related to known circoviruses is associated with porcine dermatitis and nephropathy syndrome and reproductive failure. J Virol 91.

50. Zhang HH, Hu WQ, Li JY, Liu TN, Zhou JY, Opriessnig T, Xiao CT. 2020. Novel circovirus species identified in farmed pigs designated as Porcine circovirus 4, Hunan province, China. Transbound Emerg Dis 67:1057-1061.

51. Stewart ME, Perry R, Raidal SR. 2006. Identification of a novel circovirus in Australian ravens (*Corvus coronoides*) with feather disease. Avian Pathol 35:86-92.

54. Patterson QM, Kraberger S, Martin DP, Shero MR, Beltran RS, Kirkham AL, Aleamotu'A M, Ainley DG, Kim S, Burns JM, Varsani A. 2021. Circoviruses and cycloviruses identified in Weddell seal fecal samples from McMurdo Sound, Antarctica. Infect Genet Evol 95:105070.

55. Bolatti EM, Viarengo G, Zorec TM, Cerri A, Montani ME, Hosnjak L, Casal PE, Bortolotto E, Di Domenica V, Chouhy D, Allasia MB, Barquez RM, Poljak M, Giri AA. 2022. Viral metagenomic data analyses of five new world bat species from Argentina: identification of 35 novel DNA viruses. Microorganisms 10.

56. Cerna GM, Serieys L, Riley S, Richet C, Kraberger S, Varsani A. 2023. A circovirus and cycloviruses identified in feces of bobcats (*Lynx rufus*) in California. Arch Virol 168:23.

57. Landrau-Giovannetti N, Subramaniam K, Brown MA, Ng T, Rotstein DS, West K, Frasca SJ, Waltzek TB. 2020. Genomic characterization of a novel circovirus from a stranded Longman's beaked whale (*Indopacetus pacificus*). Virus Res 277:197826.

58. Tokarz R, Lipkin WI. 2021. Discovery and surveillance of tick-borne pathogens. J Med Entomol 58:1525-1535.

59. Rinder M, Schmitz A, Peschel A, Korbel R. 2015. Complete genome sequence of a novel circovirus from zebra finch. Genome Announc 3.
